# Introductory Lectures and Addresses on Medical Subjects; Delivered before the Medical Classes of the University of Pennsylvania

**Published:** 1860-05

**Authors:** 


*Introductory Lectures and Addresses on Medical Subjects; delivered chiefly before the Medical Classes of the University of Pennsylvania.* By George B. Wood, M.D., LL.D., President of the American Philosophical Society; President of the College of Physicians of Philadelphia; Professor of the Theory and Practice of Medicine, and of Clinical Medicine, in the University of Pennsylvania, &c. 8vo, pp. 460. J. B. Lippincott & Co., Philadelphia.

But few men in America have done more to advance medical science, and to endear themselves to the profession, than has George B. Wood, of Philadelphia. For many long years he has been a laborious practitioner, a lecturer and teacher of medicine, and yet his pen has been by no means idle. To attend to all the duties of a laborious practice, to keep one's self thoroughly posted in the literature of his profession, and to lecture daily, for four months in the year, to large classes, require no small demand upon one's physical and mental energies. When, in addition to all this labor, a man writes many large volumes, of unusual merit, we must ascribe to him superior industry and talent. The United States Dispensatory, Wood's Practice of Medicine, and his Therapeutics and Pharmacology, are, perhaps, the best works of their kind in the English language.

The volume before us, and for which we are indebted to the publishers, consists of nineteen lectures or addresses, upon various subjects connected with medicine, and were chiefly introductory to yearly courses of lectures, delivered in the University of Pennsylvania.

To the many students of this time-honored University, the volume will be particularly interesting as a remembrancer of former years, when hope was big with honors and emoluments in anticipation.

Though the volume before us may add but little to our store of practical knowledge required by our every-day duties, yet it is none the less interesting, or less deserving of being read. It is full of good cheer, strengthening our resolutions of faithfulness, and encouraging us on to higher and nobler efforts. It is thus dedicated: "To the medical graduates of the University of Pennsylvania, from the spring of 1836 to that of 1860, inclusive, before whom were delivered, and in whose behalf were prepared, most of the following discourses, this volume is inscribed, as a memorial of the many agreeable, and, may I not say, profitable hours, they and I have spent together, and of the affectionate interest with which I continue, and, so long as life may last, shall ever continue to regard them.—George B. Wood."

The volume is well printed, on beautiful paper, and neatly bound in muslin. O. C. G.

